# The Inverse Response Law: Theory and Relevance to the Aftermath of Disasters

**DOI:** 10.3390/ijerph15050916

**Published:** 2018-05-04

**Authors:** Suzanne Phibbs, Christine Kenney, Graciela Rivera-Munoz, Thomas J. Huggins, Christina Severinsen, Bruce Curtis

**Affiliations:** 1 School of Health Sciences, Massey University, Palmerston North 4442, New Zealand; 2 Joint Centre for Disaster Research, Massey University, Wellington 6140, New Zealand; C.Kenney@massey.ac.nz; 3 Department of Public Health, Otago School of Medicine, Wellington 6021, New Zealand; gracielariveramunoz@gmail.com; 4 Joint Centre for Disaster Research, Massey University, Wellington 6140, New Zealand; T.J.Huggins@massey.ac.nz; 5 School of Health Sciences, Massey University, Palmerston North 4442, New Zealand; C.A.Severinsen@massey.ac.nz; 6 Department of Sociology, University of Auckland, Auckland 1010, New Zealand; b.curtis@auckland.ac.nz

**Keywords:** inverse care law, inverse response law, disaster risk reduction, social inequalities

## Abstract

The Inverse Care Law is principally concerned with the effect of market forces on health care which create inequities in access to health services through privileging individuals who possess the forms of social capital that are valued within health care settings. The fields of disaster risk reduction need to consider the ways in which inequities, driven by economic and social policy as well as institutional decision-making, create vulnerabilities prior to a disaster, which are then magnified post disaster through entrenched structural differences in access to resources. Drawing on key principles within the Inverse Care Law, the Inverse Response Law refers to the idea that people in lower socio-economic groups are more likely to be impacted and to experience disparities in service provision during the disaster response and recovery phase. In a market model of recovery, vulnerable groups struggle to compete for necessary services creating inequities in adaptive capacity as well as in social and wellbeing outcomes over time. Both the Inverse Care Law and the Inverse Response Law focus on the structural organisation of services at a macro level. In this article, the Inverse Care Law is outlined, its application to medical treatment following disasters considered and an explanation of the Inverse Response Law provided. Case studies from recent disasters, in London, New Zealand, Puerto Rico and Mexico City are examined in order to illustrate themes at work relating to the Inverse Response Law.

## 1. Introduction

Tudor Hart’s Inverse Care Law [1] refers to the idea that people who require the most care actually receive the least and to a lesser standard. The law considers how health care markets create inequities in access to services through privileging certain groups and disadvantaging others. The Inverse Care Law draws attention to the role of policy makers in influencing the distribution and quality of health care and related infrastructure. The law may also be applied in the context of access to health care in the weeks and months following a disaster as people who are most in need of medical services are least likely to receive them [2].

In the field of disaster risk reduction, consideration must be given to the ways in which inequities create vulnerabilities prior to a disaster [3]. These inequities are driven by economic and social policy as well as institutional decision-making. The resulting vulnerabilities are then magnified post disaster through systematic differences in terms of access to resources. Drawing upon the key public health principles within the Inverse Care Law, this paper proposes an Inverse Response Law that relates to the social construction of disaster risk as well as access to resources in the aftermath of a disaster event. The Inverse Care Law is explained and the Inverse Response Law illustrated through four case studies: the 2017 Grenfell Tower disaster in London, the 2016 Kaikoura earthquake in North Canterbury, New Zealand, Hurricane María in Puerto Rico, 2017, and early warning systems surrounding the Central Mexico Earthquake of 2017.

As disasters are “increasing in frequency, scale, cost and severity” [4], impacts on communities are exacerbated. Yet, there is a poverty of theory within the area of disaster studies with little development occurring over the past 50 years [4,5,6,7]. Research in the area of disasters has been criticized for remaining ‘silent on the issue of political power, for being piecemeal, isolationist and theoretically stagnant’ [4] (p. 5). Disasters provide unique opportunities for the development of theory precisely because they reveal social arrangements and lay bare forms of structural violence through surfacing prevailing forms of privilege as well as marginalization [4]. Several empirical studies have documented the impacts of disasters on human populations [8,9] with research showing social regularities in the patterning of vulnerability across time and space [4,5]. According to this research, vulnerable and marginalized communities are more likely to be impacted, with victims reflecting the established structural intersections of age, gender, ethnicity and social class [4,6]. However, theoretical explanations that make sense of these ‘facts’ are scarce. The Inverse Response Law is a mid-range sociological theory that enables scholars to explore the social patterning of vulnerability within social systems as well as its upstream drivers. Current disaster response strategies do not recognise dynamics constituting the Inverse Response Law as contributing substantially to inadequate service provision post-disaster. The authors contend that it is the communities with fewer resources that have ongoing difficulties accessing services in the disaster recovery phase, and that the proposed Inverse Response Law recognises the role of structural inequalities in effects of disasters on communities. An argument is advanced that attention to the workings of the Inverse Response Law in hazard mitigation and preparedness actions, will lead to recognition by disaster risk reduction stakeholders that communities that are socially and economically deprived prior to a disaster are most vulnerable. Targeting and resourcing a wider-ranging response could then ameliorate the existing lack of basic resources, such as poorly maintained infrastructure, to build resilience and decrease dependency on governmental and NGO sectors in the aftermath of disaster.

## 2. The Inverse Care Law

The Inverse Care Law refers to the idea that “[t]he availability of good medical care tends to vary inversely with the need for it in the population served” [1] (p. 405). That is, those who require the most care actually receive the least and to a lesser standard. The Inverse Care Law eschews a focus upon the faults and failings of individual health professionals and/or patients to illustrate the way in which the structural organisation of health services as well as local population characteristics impact upon service location, the number of practitioners available, access to health care and the quality of health care provision [10]. It looks upstream to consider how health care markets and/or government policy antecedents influence the distribution of health care resources as well as access to health care services including the key drivers of inequitable access such as poverty and/or discrimination. Reflecting on the effect of market forces on health care, for example, Tudor Hart argued that “no market will ever shift corporate investment from where it is most profitable to where it is most needed” [11] (p. 252). The Inverse Care Law originally focused upon inequalities in access to health services within an open market, however these patterns have also been observed in countries where health care is funded by the State [11]. The emphasis on inequitable access to health care associated with market provision of health services within the Inverse Care Law has become more relevant as public health services introduce dehumanizing neoliberal principles associated with competition, efficiency and effectiveness into service delivery [12].

A second aspect of the Inverse Care Law is the indication that individuals with higher levels of social and cultural capital tend to receive better care. A cultural alignment between service providers and middle class service users in the public policy arena results in the normalisation of middle class needs and demands in policy making and delivery processes [13] such as those involved in health care provision. Health care services do not embrace the kinds of skills, knowledge and networks valued by people from lower socio-economic groups creating unnecessary obstacles to care. In contrast, middle class people are more critical, they have the soft knowledge necessary to access resources, to understand the significance of information and how it impacts upon them [13]. The presence of middle class cultural capital in policy making and service delivery processes instigates a cumulative advantage whereby higher socio-economic groups accessing health care tend to receive longer consultations, superior information and advice as well as higher rates of onward referral [10,13].

Differential access to health services increases inequalities in health as disadvantaged groups fail to accrue the public health benefits of medical treatment [11]. The cost of recruitment for such treatment, as opposed to poverty and discrimination, is used to ‘explain away’ the differential uptake of services within and between groups of people within the same population. The law of diminishing returns highlights that the effort and cost involved in recruiting the remaining 20 percent of a population into medical research is higher than the effort and cost involved recruiting the first 80 percent [11]. For this reason, limits are imposed on recruitment of ‘hard-to-reach’ populations such as those affected by severe economic deprivation. Under-representation of deprived populations in medical research becomes important when research is in the area of service provision and acceptability. Research evidence and primary health care programmes aimed at preventing disease reflect the Inverse Care Law whereby populations with the greatest degree of health care need are least likely to participate [11]. The law of diminishing returns suggests that when health care interventions are rolled out with the same resources across the population then interventions aimed at lower socio-economic groups will achieve less [11] thereby perpetuating disparate access to quality health care over time.

### The Inverse Care Law Following Disasters

The Inverse Care Law suggests that ‘people who are most in need of medical services are least likely to receive the care that they need in the weeks and months following a disaster’ [2] (p. 12). There are large social inequalities in the patterning of vulnerability and the distribution of resources post disaster [14,15]. Communities with fewer resources are at greater risk post-disaster and have ongoing difficulties with routine access to health care and social services in the recovery phase compared to those sectors of the population with better health and access to health care [16,17]. The disaster literature, for example, has identified reduced access to health care facilities [2,18,19] as well as social inequalities in access to clinical treatment among poor and medically underserved people living in a disaster zone [16,17]. Research in this area has specifically linked the Inverse Care Law to access to housing [20], health care [2], and support [21].

The sudden increase in the demand for emergency medical services in the immediate aftermath of a disaster is termed the primary or initial surge [16]. During the primary surge the health care system is immediately overwhelmed with injuries and acute illness needs. Secondary surge refers to the sudden increase in the need for long-term health care services for incident-related chronic disease in the months and years after the initial impact [16]. Fewer health care resources, such as medical centres, health professionals, specialist services, pharmacies and medical equipment, are generally available to people in the aftermath of disasters [2,16]. Access to health care services may be further restricted by the loss or transfer of medical facilities outside of the disaster zone resulting in exacerbations in pre-existing conditions due to a lack of continuity of care and delays in chronic disease management [2,16]. Already poor and/or medically underserved communities are disproportionately affected by health disparities and are therefore more likely to be impacted by reduced access to health services within a disaster zone. Prior to a disaster, decisions made around planning for and funding an upsurge in demand for services in areas as diverse as cardiovascular disease [22], mental health [22], and aged care [23] may help to mitigate a range of poor health outcomes immediately post-disaster and in the recovery phase.

The Inverse Care Law draws attention to the underlying social conditions that influence the provision of health services as well as access to health care resources. The new emergency management framework [24] also pays attention to the social determinants of vulnerability, including how inequities in the distribution of resources, wealth and opportunity create disparities outcomes in the aftermath of disaster [25]. Principles within the Inverse Care Law, relating to the structural distribution of resources, as well as key drivers of inequities in access to services is therefore relevant for disaster risk reduction, preparedness, planning, resourcing, response and recovery.

## 3. The Inverse Response Law

Drawing upon the principles within the Inverse Care Law, we propose an Inverse Response Law illustrating differential access to emergency services and supplies in the aftermath of a disaster. The Inverse Response Law suggests that those people in lower socio-economic groups, who tend to be disproportionately impacted in a disaster, tend to receive the least help and to a lesser standard. The Inverse Response Law draws attention to how markets create vulnerabilities through influencing the distribution of social and economic resources prior to a disaster as well as the provision of services during the recovery. It considers how structural inequalities increase susceptibility to disaster as well as the role of public policy in creating or ameliorating risk. It reflects upon how social and cultural capital, in the form of personal networks and skills, influences disaster preparedness activities as well as access to resources post impact. The Inverse Response Law also recognises that when disaster preparedness activities, as well as the provision of resources post impact and in the recovery, are rolled out with the same resources across the population, then programmes aimed at lower socio-economic groups will be less effective. Finally, the Inverse Response Law focuses upon the way in which the structural organisation of emergency management resources and infrastructure influences the capacity of emergency service personnel to respond.

### 3.1. Revisiting Heatwave

Klinenberg’s [18] detailed social autopsy of the 1995 Chicago heatwave may be revisited in relation to key principles within the Inverse Response Law relating to market forces, the structural distribution of resources, inequity of access and inequalities. Heatwaves cause more deaths in the United States than any other extreme meteorological event. Unlike other natural hazard events, such as an earthquake or a category five cyclone, heatwaves leave the emergency management resources and infrastructure in place thereby illustrating how a city’s social environment contributes to a disaster as well as its institutional capacity to respond. During the 1995 Chicago heatwave temperatures exceeded 100 degrees Fahrenheit (37 degrees Celsius) over six consecutive days resulting in an excess mortality of 739 city residents during the week of 14th to 20th of July [18]. According to Klinenberg, the victims of the Chicago heatwave were “primarily social outcasts—the elderly, the poor, and the isolated… Silent and invisible killers of silenced and invisible people” [18] (p. 17).

### 3.2. Institutional Barriers Disproportionately Impacted the Poor

The Chicago heatwave was characterised by institutional barriers to an effective response. The city ignored its own guidelines for declaring a hot weather emergency and the local media ignored meteorological service warnings that a deadly heatwave, that had already caused deaths in other jurisdictions, was on its way [18]. The Fire Department, which was charged with overseeing an emergency response, had no centralised means to monitor demand for emergency services and no mechanism in place to automatically trigger a crisis response. The Fire Department, which managed the ambulance service at the time, was unaware of the rapid increase in call-outs or that between July 13 to July 16 a total of 23 hospitals were periodically on bypass and that 18 hospitals were on bypass at the same time. The Chicago Health Department was also not informed of the crisis in hospital-based emergency care within the city. When the scale of the emergency was belatedly identified, fire engines were enlisted to provide extra cover, however officials failed to equip them with the means to cool people down and potentially save lives. When people did reach medical care, health officials and physicians had insufficient training in managing heat-related illnesses [18].

Applied here, the Inverse Response Law considers how first responders are impacted by the structural organisation of emergency management resources and infrastructure. The city was unable to respond appropriately because it failed to recognise the encroaching threat posed by the heatwave or to respond appropriately to front-line emergency service staff who were raising concerns about the emerging crisis [18]. The circumstances that led to this failure were set in place prior to the disaster. Many people died because of these institutional failures, and those who were impacted were mainly poor and elderly African Americans [18]. In this example of the Inverse Response Law, upstream factors related to the structural organisation of disaster risk communication, monitoring of service demand and training to manage heat-related casualties prior to the disaster, created barriers to an effective emergency response ensuring that the downstream response was inadequate compared to need.

### 3.3. The Inverse Care Law: Inequities in the Geographical Distribution of Services

The Inverse Care Law, which considers how the market influences the location of health care resources, may be identified in Chicago through inequities in the geographical distribution of health care facilities and police officers. Klinenberg [18] notes that medical services were concentrated in the wealthiest areas of Chicago and that during the heatwave crisis the emergency departments in many of the hospitals on the south side of Chicago were no longer admitting patients. Incidents of life-threatening heat-related illness were concentrated among residents living on the poorer south side, these people had the greatest need but experienced numerous obstacles to care. During the emergency only 59 ambulances and paramedic teams were available within the region of Cook County in which Chicago city is located. The Fire Department and the Health Department failed to recall off-duty staff and emergency staff failed to requisition ambulances and paramedics from surrounding areas in order to lift the number of available ambulances to 145. The inadequate supply of ambulances and paramedic staff combined with longer travel times to get people into a hospital that was still admitting patients increased overall waiting times for emergency response teams [18]. An inverse distribution of police officers was also noted, as the same number of officers were allocated to each city ward regardless of the crime rate. This policy meant that police officers in low-income high crime areas were overworked and unable to engage in preventive and community policing activities [18]. Community-based police work enables officers to have prior knowledge of the community which would facilitate identification of individuals who were potentially vulnerable to heat related illnesses prior to the crisis. In the context of the Chicago heatwave, this knowledge had the potential to save lives.

### 3.4. Disadvantaged Communities had Greater Exposure to Disaster Risk

The Inverse Response Law draws attention to how markets create vulnerability to disaster through influencing the distribution of social and economic resources. Disaster risk is historically, geographically and socially patterned [26,27] and disproportionately prevalent in disadvantaged communities [3,18,26,27,28,29,30,31]. People who are poor and disadvantaged are more likely to be impacted [18,32] have fewer resources to fall back on in a disaster [29,32] and are more likely to experience adverse outcomes over time [25,32,33]. The patterns of death resulting from the Chicago heatwave reflected existing patterns of inequality within Chicago at the time. The majority of the victims (73%) were over 65 years of age with African Americans having the highest proportional death rates of any ethnic group. Men were more than twice more likely to die than women [18]. The geography of vulnerability mapped onto existing area-based inequalities, with low income, African American and violent areas of the city enduring the greatest impacts.

### 3.5. The Impact of Policy

Inequities driven by public policy and institutional decision-making before a disaster act upon the entire social system to aggravate systematic differences in outcomes among vulnerable populations post disaster [2]. Public policy may undermine community resilience through increasing social and economic disparities, exacerbating homelessness, eroding social connectedness and reducing trust. The normalisation of middle class needs and demands in policy making and delivery processes [13] is illustrated in the social distance between state agents and residents of stigmatised black neighbourhoods in Chicago. A climate of fiscal austerity, combined with the outsourcing of public services to private providers, resulted in significant staff reductions in city agencies responsible for health, human services and housing. Financial constraints on social service provision also made it difficult for administrators to request additional resources [18]. These policy decisions increased vulnerability through lessening the safety net for the elderly as well as residents within disadvantaged communities. Public housing administrative staff, for example, contacted vulnerable residents during the heatwave, residents in low income or temporary housing that was provided by the private sector did not receive this service [18]. In this example, social distance between people who are dependent upon the state and legislators is intensified through the imposition of middle class concerns, associated with cost-cutting and the outsourcing of public services, onto social service provision. The Inverse Response Law thus is illustrated through the way in which changes in public policy help to create the particular social conditions that underpinned the patterning of deaths during the Chicago heatwave.

Klinenberg’s work draws attention to the way that disasters are socially constructed, occurring within the social, cultural and historical context of neighbourhoods, social service systems and government programmes. Key principles within the Inverse Response Law may be used to think about disasters that have happened more recently.

## 4. The Inverse Response Law and the Social Construction of Disaster Vulnerability

The examples provided in the following sections illustrate the Inverse Response Law through focusing upon the structural conditions that influence the social patterning of vulnerability as well as disaster risk. In the first example, the immediate social service response to the Grenfell Tower disaster in London provides an illustration of a key principle within the Inverse Response Law whereby disadvantaged communities have greater need but received the least help and to a lesser standard. Grenfell Tower is also an example of how the structural organisation of public housing policies contributed to the disaster through exacerbating inequalities and transferring risk to council housing residents. In the second case study, the 2016 earthquake in Kaikōura, New Zealand, we consider the role of social capital in a disaster through exploring challenges for rurally isolated communities in having a voice and accessing resources post-disaster. The final two case studies explore the ways in which disaster risk is disproportionately prevalent in disadvantaged communities. The case study of Hurricane María, which devastated Puerto Rico in 2017, explores how vulnerability is constructed through the historical positioning of the region as a US dependency as well as through contemporary federal government policies which continue to negatively impact the island. The final case study considers the performance of, as well as inequities in access to, Mexico City’s early warning system following the 2017 Mexico City earthquake. We highlight the inequities in access to the city’s early warning systems and argue that social inequalities in the placement of the systems have the potential to exacerbate inequalities in outcomes post-disaster. In all of these examples, vulnerability can be traced back to deliberate decisions that were made by policy makers and funding agencies—and in this regard the negative human impacts of the disasters may be regarded as entirely social.

### 4.1. Grenfell Tower, London, 2017

On the 14th of June 2017, 71 people were killed in the Grenfell Tower disaster and hundreds left homeless. While emergency services and the community rallied to provide support to survivors of the fire, as well as surrounding residents who were temporarily evacuated from their homes, at local and national government levels an official response to the disaster was lacking. The Inverse Response Law draws attention to how structural inequalities in the London housing market creates vulnerabilities as well as the role of public housing policy in producing or ameliorating risk. Inequalities in the distribution of social service resources following the Grenfell Tower disaster also illustrate how the macro organisation of formal emergency management resources and infrastructure influence the capacity of state agencies to respond. At the opening of Parliament on the 21st of June 2017, Prime Minister Theresa May recognised this social service failure and formally apologised to the community:

Let me be absolutely clear: the support on the ground for families in the initial hours was not good enough… That was a failure of the State, local and national, to help people when they needed it most…

[34]

While the Grenfell Tower disaster exposed a lack of disaster response capability at the national level, the local council also failed to step up to fill the void. Grenfell Tower is located in the Royal London borough of Kensington and Chelsea. The borough is one of the most unequal in London, a place where extreme wealth and poverty sit side by side. In 2015, Grenfell Tower was rated as among the top 10 percent of deprived areas in England. In contrast, the constituency of Kensington, which makes up most of the local authority, is the wealthiest in England [35]. At the time of the fire, the Kensington and Chelsea Council was one of the wealthiest local councils in the country with £274 million in reserves [36].

At the first council meeting since the fire, the leader of the Kensington and Chelsea council, Nick Paget-Brown, acknowledged, “media criticism for a slow reaction to the fire, non-visibility and for failing to invest in North Kensington” [37]. Paget-Brown accepted the condemnation of the council’s response to the fires and said he would “apologise for what we could have done better” [37].

In the cosmopolitan, international city of London, in the midst of extreme wealth the Grenfell Tower disaster provides a perverse illustration of the key principles within the Inverse Response Law—that those who require the most help are the least likely to receive it. The Grenfell Tower disaster was not a just a failure of the emergency response, illustrated by inadequate social service provision in the aftermath, but a structural failure in the organization of social housing at a macro level. The principle within the Inverse Care Law, that people in lower socio-economic groups tend to obtain lesser services, receive less attention and have poorer quality facilities appears to also apply to the Grenfell Tower disaster. Media reports that followed the disaster [38,39] noted that residents had repeatedly raised fire safety concerns with the Kensington and Chelsea tenant management organisation including: the absence of an alternative fire escape route, the placement of unprotected gas pipes in the main stairwell, the lack of a sprinkler system, and problems with the accumulation of rubbish within the building [38]. The Grenfell Tower tenant management organisation also declined a request by residents for an independent fire safety audit. In November of 2016, the Grenfell Action Group warned that “only a catastrophic event will expose the ineptitude and incompetence of our landlord” [38].

Tudor Hart’s [12] observation that in health care corporate investment tends to be skewed towards profitable jurisdictions, creating barriers to access for those in lower socio-economic groups, may also be applied to the area of housing. In 2010, the UK government cut local authority budgets by 40 percent, these reductions in public service spending encouraged local councils to accept low cost tenders for the maintenance of the region’s social housing stock. At the same time, the transfer of financial risk, associated with building new council housing, from the public service to private contractors reduced council oversight and incentivised cost cutting in order to deliver profits to shareholders. At the national level, fire service cuts resulted in 11,000 fewer fire fighters throughout the UK as well as reductions in the number of building control inspectors and fire safety audits. These changes potentially worked to erode the culture of safety at government and corporate governance levels. Government legislation passed in 2005 shifted responsibility for fire safety from the fire brigade to local councils [40] potentially introducing a conflict of interest when fire safety audits were conducted on council owned property. Echoing the Chicago heatwave, the patterns of death resulting from the Grenfell Tower disaster reflected existing social inequalities within the city. Inequities driven by public policy increased the vulnerability of Londoners who are reliant on social housing, contracting out of services and cost cutting resulted in savings accrued through the transfer of fire safety risk to the poorest residents of the city. Thus, the social conditions that led to the deaths of 71 people were set in place prior to the disaster. People died because of a range of institutional failures created through conservative neoliberal approaches to public policy, and those impacted were mainly poor, immigrants and/or refugees.

### 4.2. Kaikōura Earthquake, New Zealand, 2016

Since 2010, New Zealand has experienced a series of significant seismic hazard events, which have wrought widespread social, economic, and environmental damage. The most recent event, the Kaikōura earthquake, occurred on the 14th of November, 2016, measured 7.8Mw, and was centred 15 km north of the town of Culverden in North Canterbury [41]. Although the broader North Canterbury, Marlborough and Wellington regions experienced major damage primarily to buildings and transport infrastructure, Kaikōura and the wider Hurunui districts were the most severely impacted areas [42].

A sequence of strong aftershocks, in combination with the impacts of a severe weather system, compounded initial regional damage causing more than 80,000 landslides [43,44] and massive damage to lifelines, utilities, and transport infrastructure along the North East Coast of the South Island. Although electricity and communications outages were largely resolved in the first 48 hours following the earthquake [45], land transport was completely disrupted. Coastal communities were somewhat accessible by sea, and the main coastal community, Kaikōura, acted as a hub for the government’s emergency management response to the earthquake. Access routes through the Hurunui district to the town of Hanmer Springs and across the Lewis pass to Springs Junction were also established within 24 h after the earthquake [46]. However, some remote farming communities in the Hurunui and Kaikōura districts were totally cut off, or only accessible by helicopter, and thus comparatively disadvantaged in terms of initial access to recovery resources.

According to the Inverse Response Law communities with fewer resources have ongoing difficulties accessing services in the disaster recovery phase. In this case study, the role of social capital in disasters is considered through exploring the challenges that rurally isolated communities faced accessing resources following the 2016 Kaikōura, New Zealand earthquake. Inland rural communities in North Canterbury bore the brunt of the M7.8 earthquake, however their needs were largely ignored in government funded recovery programmes. Traumatised rural communities in Marlborough at the top of the South Island, that were severely impacted by the 2016 Kaikōura earthquake and since 2013 had also experienced two shallow earthquakes of M6.5 and M6.6 as well as numerous aftershocks, were largely absent from public discussions of the disaster. Thus, an argument is made that post-disaster both the media and policy makers were captured by the concerns and demands of people affected in the less severely damaged urban areas of New Zealand’s capital city Wellington who had easier geographical access to, as well as networks into, both the fourth estate and the corridors of power.

New Zealand’s emergency management response to the earthquakes involved multiple international, state and non-governmental agencies. A modular Coordinating Incident Management System (CIMS) was introduced in 2004 as a nested framework that connected key response stakeholders at local, regional and national levels in the event of a disaster [47]. This system activated immediately following the Kaikōura earthquake bringing together government actors, emergency managers, as well as providers of critical infrastructure, relevant science, health and welfare services in an integrated decision-making structure that determined and operationalised the formal response to the earthquake. The Ministry of Civil Defence and Emergency Management led the response and prioritised the provision of essential resources (water, food, fuel) to isolated communities [48]. New Zealand’s Defence Forces in partnership with naval allies from the United States of America, Australia, and Canada evacuated several hundred tourists and residents from Kaikōura as well as conducted flight reconnaissance of environmental and infrastructure damage [49]. The local iwi (Māori tribe) Ngāti Kuri opened their marae (Māori community centre) Takahanga as a shelter and welfare hub and with support from the main Iwi body Te Rūnanga o Ngāi Tahu and local Kaikōura residents, provided shelter, meals, clothing, hot showers, and evacuation transport during the first 10 days after the earthquakes [50]. As health services support was also considered a priority, the Canterbury District Health Board deployed two medical officers of health, four occupational health officers, and 20 clinical staff to Kaikōura to provide support for the local general practitioner, pharmacist, and allied health professionals [51]. Although there were few related fatalities, approximately 580 local residents have reported earthquake-related injuries to date [52], contributing to an increased demand on health, accommodation and other social support services as local communities recover from the earthquakes. The ability of some local communities to physically access these services has been restricted in part due to unavoidable geographical, infrastructural and, in some instances, psychosocial factors. More broadly, the availability of resources essential to the recovery process has been shaped by a range of governance, economic and political externalities.

In the Civil Defence Emergency Management Act (2002) [53] recovery from an earthquake event is defined as the co-ordinated efforts and processes used to bring about the immediate, medium-term, and long-term regeneration and enhancement of a community following an emergency. Thus, collaborative development and implementation of recovery strategies for the Hurunui and Kaikōura regions has involved a range of Government ministries, non-governmental organisations, iwi, private stakeholders (e.g., insurance companies), as well as local authorities or district councils. Under the (2002) [53] Act, the local Hurunui and Kaikōura District Councils have specific roles; assessing and monitoring the needs of communities affected by the earthquakes and coordinating local recovery initiatives. Councils are also required to implement measures that: restore and enhance the built, natural, rural, social, and economic aspects of affected communities; ensure the cultural and physical well-being of individuals and communities; support Government and non-government organisations to work together and enable communities to participate in recovery planning. In order to address these responsibilities, the Kaikōura and Hurunui District Councils established recovery management teams that coordinated and oversaw the implementation of recovery initiatives. However, the effectiveness of recovery approaches was heavily underpinned by access to resources and funding from the New Zealand government, the New Zealand Earthquake Commission as well as local agents such as the Canterbury District Health Board. If disaster governance agents prioritise resource provision with regard for the economic, social and cultural capital of communities affected by earthquakes, then the response to impacts of the Kaikōura earthquakes on Wellington, the capital city and seat of government in New Zealand, may constitute a case in point.

As a result of earthquake damage, approximately 11 percent of urban office space in Wellington was closed or vacated, inner city residents in several apartment blocks were evacuated, while several city blocks were cordoned off from road and pedestrian traffic [54]. Significant levels of liquefaction were also noted along sections of the Wellington waterfront to which access was subsequently blocked off, and the Port of Wellington’s gantry was disabled, resulting in the cessation of container shipping for several months [55]. However, the majority of urban residents were not displaced from their homes. With the exception of some parts of the Port of Wellington, local transport and utilities infrastructure remained intact, demolition of damaged buildings was negligible, and health and social services were not compromised. Yet, in the aftermath of the Kaikōura earthquakes, government, local authority, private sector and scientific attention has intensified around future proofing the earthquake resilience of Wellington [56].

In contrast to the focus on Wellington, the comparatively greater recovery needs and aspirations of North Canterbury communities have become less prominent in the public domain. Three months after the earthquake, media reports in early 2017 noted that despite an increased demand for health services in both regions, Kaikōura and Hurunui were excluded from the post-Canterbury Earthquakes’ ‘All Right’ psychosocial recovery programme [57]. Moreover, online news reports also stated that the Canterbury District Health Board had received inadequate Kaikōura earthquake mental health funding from the Ministry of Health [58]. Following the change of government in 2017, mental health funding shortfalls in the Canterbury region have been under review, with the Government recently announcing a $28 million dollar increase in mental health support for Canterbury children [59]. However public perceptions that the health needs of Kaikōura were being privileged at the expense of the Hurunui region, drew strong criticism from the wider community, exemplified in the words of Canterbury District Health Board Deputy Chairperson Sir Mark Solomon, who stated: Quake-hit Hurunui residents felt “forgotten by the Crown” [60]. While, the ‘All Right?’ psychosocial recovery programme (developed following the 2011 Canterbury earthquakes) has since been rolled out to Kaikōura and Hurunui district residents [61] and further government financial support for enhancing health services in both communities has been accessed, it has taken a considerable length of time to access funding that is still deemed as inadequate [62].

Runkle [2] proposes that in the aftermath of a disaster, communities with the greatest needs have difficulty accessing essential services and support, and that the comparatively inequitable access to assistance correlates directly to low socio-economic status. Prior to the earthquakes, local economies in the Kaikōura and Hurunui regions were thriving with economic activities centred on primary industry and tourism [63]. However, Kaikōura has a larger population, and prior to the earthquake, was an internationally renowned marine eco-tourism destination, whereas the Hurunui region was a common holiday destination for primarily local Cantabrians. Subsequent to the earthquakes, both regional economies were severely affected. A substantial reduction in tourism numbers resulted in staff layoffs and business closures primarily in Kaikōura [64]. Disrupted infrastructure negatively impacted the wellbeing of farm stock as well as milk collection and transport [65], while closure of coastal fisheries resulted in the loss of $23 million dollars from harvesting crayfish each year and a further $1.5 million from annual paua (abalone) exports [66].

The immediate reduction in economic viability, enhanced geographical isolation, and related social vulnerabilities experienced by the North Canterbury communities may be juxtaposed by the situation in Wellington. The city is centrally located within the country and widely accessible by air, sea and land transport links. As the seat of Government, the city has a large population, is a major hub for employment, education, and corporate business and is developing an international reputation for tourism, film production, and the wider creative arts. Therefore, in comparison with the Kaikōura and Hurunui regions, it may be argued that over time Wellington has accrued significantly more economic, cultural, and social capital. The disparities in accumulation of different forms of capital between the regions can be benchmarked against the levels of resources made available and speed with which ongoing recovery issues have been addressed in both regions. As an exemplar, Civil Defence delays purportedly held up the assessment of 3,500 damaged homes at the epicentre of the earthquake, which resulted in building evaluations that should have taken two weeks, taking almost twice as long [67]. One rationale for the delay provided by the Canterbury CDEM Group was that there were insufficient building inspectors in the region to assist the Hurunui District in conducting assessments [67]. However, causative factors for this delay were systemic and included organisational decisions which resulted in the relocation of Canterbury and CDEM building inspectors to Wellington to assist with assessment of buildings in the central business district. Broadly speaking, subsequent to the Kaikōura earthquake, the attention of central government, the Wellington City Council and national science research has remained focused on how Wellington will mitigate ongoing seismic risks as well as cope in the event of a major earthquake [68]. These contrasting responses to the impacts of the Kaikōura earthquake clearly showcase how, as Fothergill and Peek [15] suggest, systemic and social processes contribute to reproducing social inequity in the distribution of resources following a disaster.

This case study has drawn attention to the ways in which the Kaikōura earthquake experiences and concerns of rural communities in North Canterbury have largely been silenced, while the earthquake impacts and effects of future seismic events on New Zealand’s capital city have been privileged by diverse stakeholders including central and local government, and the media. The inequity highlighted provides a clear exemplar of how the Inverse Response Law has fully applied in the context of the Kaikōura earthquake.

### 4.3. Puerto Rico—Hurricane María, 2017

On the 20th of September of 2017, Hurricane María swept through Puerto Rico as a Category 5 tropical cyclone [69]. The cyclone occurred within weeks of a series of major hurricanes hitting Puerto Rico, other Carribean islands, and the US [70]. These storms caused devastation not seen in the region for over 100 years [71]. After Hurricane María struck, the newly elected Governor Ricardo A. Roselló Nevárez described the situation in Puerto Rico as “apocalyptic” [72] and four weeks after Hurricane María hit, the circumstances were described as “nightmarish” [73] as human need was still outstripping the most basic life preserving resources available. At this time, there were 64 reported direct deaths and upwards of 1052 preventable deaths [74], one million people without safe running water, more than three million people without power, and 40 percent of cell phone towers were inoperable [75]. Before the hurricane, the population of Puerto Rico was 3.3 million people, 70 percent of whom resided in the territory’s major urban centres including the metropolitan area of the capital city of San Juan in the north, Ponce on the southern coast, and Mayagüez to the west [76]. All of these urban centres were significantly damaged by Hurricane María, with the large-scale flooding of parts of San Juan making international headlines [77].

This case study uses the principles of the Inverse Response Law relating to inequities in the structural distribution of risk as well as in access to resources and services to examine the impact of this disaster sequence on Puerto Rico, including how the island’s complex political and economic climate has impinged on the process of disaster recovery. It is argued that colonial power, inequality and economic crises have negatively influenced the structural organisation of the federal response, as well as the quality of services received by Puerto Ricans in the aftermath of the disaster.

Puerto Ricans have been US citizens since the passing of the Jones Act in 1917 [78] and, since then, the territory’s demographics have been shaped by migration to the US mainland due to the territory’s ongoing economic crisis [79]. This ‘exodus’ [80] has been compounded by the 2017 disaster which resulted in another 250,000 to 500,000 people leaving the territory for the United States mainland [81]. Those who have been most affected by Hurricane María are the ones who cannot afford to leave and can least afford to wait. The Inverse Response Law position that disaster risk is disproportionately prevalent in disadvantaged communities is illustrated here by the experiences of the most vulnerable Puerto Ricans - those who live in the flood-prone areas of the island’s rundown major cities and poorest rural towns nestled in the forested central highlands, that were left isolated after the hurricane’s eye passed right over them. As without a large scale recovery, “the island is likely to find itself back in the same place after the next big storm” [82].

The history of Puerto Rico has been marked by colonial governance, and this has shaped the political and income inequality experienced by many generations of Puerto Ricans. As a result of the Spanish-American War in 1898, Puerto Rico became a possession of the United States. The US did not grant independence to Puerto mainly due to its geo-strategic position in the Atlantic for defence purposes. In 1952, in order to quell a popular desire for self-government, the US authorised Puerto Rico to adopt a constitution. Since then, Puerto Ricans elect members to a bicameral legislature as well as a territorial Governor. The Puerto Rican government can legislate on most local issues, but not in ways that are inconsistent with US federal laws that apply as in any other US jurisdiction. Although Puerto Ricans are US citizens, they do not have the right to vote for US President or members of Congress [83]. Thus, the ambivalent federal government response to Hurricane María needs to be understood in relation to the history of US colonial rule in Puerto Rico, its disenfranchised citizenry, as well as the difficult state of the Puerto Rican economy at the time of the hurricane.

Starting in the 1930s and 1940s, Puerto Rico embarked upon large-scale economic and social development programmes, like “Operation Bootstrap” a self-help approach to economic and social reform, to transform the island’s agricultural monoculture economy to one based on heavy and light industry [84]. A special tax status treating Puerto Rico as a foreign jurisdiction to the US was introduced by Congress in 1900 with the Foraker Act [85]. This special tax status served to attract investment by making Puerto Rico government bonds and corporations established in the territory exempt from US federal income taxes [86]. However, the special provision in the US Internal Revenue Code granting this exemption to corporations based on the island [87] was rescinded by Congress in the 1990s [88], and together with free trade agreements (like the North American Free Trade Agreement, NAFTA), had a severe impact on Puerto Rico’s economic base. By and large, this top-down approach to economic and social development failed to raise all Puerto Ricans out of poverty. Currently, over 43.5 percent of those living in Puerto Rico have an income below the US Census Bureau official ‘poverty threshold.’ This makes the territory much more socially and economically deprived than Mississippi, the poorest US State [76].

As hurricanes Irma and María struck, Puerto Rico was undergoing a severe economic crisis. It was recently estimated that the recovery will cost over $95 billion USD [89], at a time where Puerto Rico’s economic prosperity was being hampered by crippling public debt. Colón-Ríos [87] stated, “[t]he crisis is in many ways connected to the special federal tax status […], which made the island particularly attractive to investors and increased in important ways the island’s ability to borrow money” through the issuing of tax-exempt government bonds [90]. However, after years of over spending and political corruption, in 2014, “Puerto Rico’s access to capital markets received a major blow […] when rating agencies downgraded Puerto Rican bonds to non-investment grade” [91]. This down-grading of capital markets was coupled with a US Supreme Court ruling that prevented Puerto Rican municipalities from accessing the federal bankruptcy process. Colón-Ríos [87] argues that, “[f]rom the perspective of the island’s economy, this was a disastrous decision; combined with Article VI, Section 8 of the Constitution of the Commonwealth of Puerto Rico of 1952, which […] states that in case of insufficiency interest on the public debt and amortization thereof shall first be paid,” before any public expenditure, including core services [87].

In light of these financial crises, in 2016 the US Congress passed the “Puerto Rico Oversight, Management, and Economic Stability Act” (PROMESA) [92]. This Act co-opted even more of the territory’s political autonomy with the appointment of an Oversight Board with extensive statutory powers over Puerto Rico’s locally elected government. The goal of this policy was to compel the territory to pay its public debt of 72 billion (USD) with Wall Street, in detriment to its other public obligations through the approval of a ‘Fiscal Plan’. The Board also has a final say in the approval of the island’s ‘Fiscal Plan’ “[which has the main objective of providing ‘a method to achieve fiscal responsibility and access to capital markets’]” [92] and must determine if the budget ‘proposed’ by the Government is ‘compliant’ with relevant fiscal priorities.” Colón-Ríos [87] explains how the PROMESA Act has profound implications for the already compromised democratic status of the territory stating that it:

“dramatically changes the ways in which political power is exercised in the island, diminishing in important ways the rule making faculties of the executive and legislative branches of government.”

The political relationship outlined between Puerto Rico and the US provides an important backdrop for understanding the application of the concept of the Inverse Response Law to the case of Puerto Rico. Indeed, from this relationship stem many of the social and economic inequalities endured by Puerto Ricans and it has shaped the disaster response and recovery process following Hurricane María.

The undemocratic Puerto Rican statutory framework gives the US Federal Emergency Management Agency (FEMA) complete authority over disaster response and recovery within the territory. The immediate response was criticised in the international media which highlighted damage to the territory’s already weakened infrastructure as well as the lack of response from FEMA and the US President. It has been reported that the federal response “has been plagued by mistakes, waste and apparent cronyism” [93]. FEMA’s struggle to handle the emergency was evident when, in a further erosion of the territory’s political autonomy, the US appointed a General to oversee a military response after the disaster [94]. The complexities of delivering aid to Puerto Rico, outside of the US mainland, were plainly summed up by President Trump at a press conference when he stated, “[t]his is an island surrounded by water, big water, ocean water” [95]. However, early on, difficulties delivering aid to Puerto Rico were compounded, not only by the geographical location of the island territory, but by the US President’s initial refusal to temporarily suspend the Jones Act of 1920 which restricts the carriage of goods from port to port within the USA to American built, owned and crewed ships [96]. The formal disaster response has been sharply contrasted by that of the Puerto Rican civil society and the diaspora which mobilised in an impressive demonstration of social solidarity [97], much of this organising taking place through social media and community outreach [98]. In his very public spat with the Mayor of San Juan, Carmen Yulín Cruz [95], President Trump omitted to acknowledge this community response when he tweeted, “[t]hey want everything to be done for them when it should be a community effort[;] 10,000 Federal workers now on Island doing a fantastic job” [99].

President Trump and his entourage visited Puerto Rico and the US Virgin Islands on the 3rd of October of 2017. Instead of highlighting the challenges facing residents, accompanying media largely focused their reports on the President’s actions including throwing paper towel rolls at a clamouring crowd [100] and the First Lady’s choice of footwear [101]. Less than 10 days later the President expressed his frustration with the situation in Puerto Rico, again on Twitter:

“Puerto Rico survived the Hurricanes, now a financial crisis looms largely of their own making. […] A total lack of […] accountability say[s] [sic.] the Governor. Electric and all infrastructure was disaster before hurricanes. Congress to decide how much to spend. […] We cannot keep FEMA, the Military & the First Responders, who have been amazing [under the most difficult circumstances] in P.R. forever!”.

[102]

In a disaster context, emphasis on the payment of public debt to private lenders over the immediate concerns of a population relates to the principle within the Inverse Response Law concerning the role of public policy in creating risk through diverting resources from those in greatest need. Despite his populist campaign message, President Trump has doubled down on this debt repayment policy, tweeting “[m]uch of the Island has been destroyed, with billions of dollars owed to Wall Street and the banks which, sadly, must be dealt with” [103]. This situation has been reflected upon by prominent authors who argue that the recovery process in Puerto Rico is an example of disaster capitalism. In the “Shock doctrine”, Klein [104] proposes that, national moments of crisis are abused by actors with special interests to impose controversial neoliberal policies, at a time of collective trauma when the citizenry is unable to mount an effective political resistance [104]. After a recent visit to the island, the author expressed that [w]e’re seeing the strategy that we’ve seen in many other disaster zones, which is exploiting that state of shock and distraction and emergency to push through a radical corporate agenda” [105]. Indeed, the extraordinary law-making powers of PROMESA and the Oversight Board have been key in pushing austerity measures [106] and neoliberal reforms after the disaster, including proposed asset sales (like the Puerto Rico Electric Power Authority, PREPA), [107] an educational reform that has closed down public schools in favour of the establishment of charter schools, [108] and a proposed labour reform that significantly limits workers’ rights [109].

Overall, the response from the Republican-led US Congress can also be considered problematic from the perspective of resource distribution within the Inverse Response Law. Of the $95 billion USD requested for disaster recovery to Congress for the 2018 US federal budget by the Governor of Puerto Rico, four months after Hurricane María struck, the short-term spending bill approved to end the US government shut down [110] only included the following:

“$4.9 billion to increase Medicaid caps for Puerto Rico and the U.S. Virgin Islands for the next two fiscal years. Also, 100% of Federal Medical Assistance Percentage will be applied to new funds. $9 billion for housing and urban development projects in Puerto Rico. $2 billion to repair Puerto Rico’s electrical grid. $1.4 billion for the Federal Highway Administration’s Emergency Relief Program is being made available for Puerto Rico and other impacted areas. Puerto Rico is also provided 100% federal cost share for damages resulting from Hurricanes Irma, and Maria for the next two fiscal years. $519 million to repair Army National Guard sites in Puerto Rico and U.S. Virgin Islands. $31 million for rehabilitation and repair Department of Labor Job Corps Centers in Puerto Rico”.

[111]

Also concerning was the Republican congressional majority’s insistence that their flagship tax reform bill (the Tax Cuts and Jobs Act of 2017) contain provisions “aimed at bringing operations and jobs back to the [US] from overseas would apply to Puerto Rico just as they would to […] any other foreign jurisdiction” [112]. This policy will result in the increased loss of American investment and employment on the island, while it is recovering from a severe economic crisis and its biggest disaster in 100 years. These additional economic and legislative blows to Puerto Rico’s economy, illustrates how the territory’s powerless political relationship with the US underlies the unequal nature of the recovery process. In summary, key principles within the Inverse Response Law are illustrated by the island’s undemocratic political relationship with the US, which is complex and long-standing. The Inverse Response Law may also explain how this unequal and undemocratic political relationship impacts upon the direction of the recovery process. It is not adequate to hope that the resilience of the Puerto Rican people is enough to help the territory ‘bounce back’ from this abyss of historical proportions. The scale of this disaster is just too great. A serious recovery plan needs to take into account the structural limits imposed on the territory by its political status as well as the many community strengths and assets available in this context. Already the territory’s unique sense of culture has allowed the Puerto Rican diaspora to mobilise in support of those back home. How the recovery proceeds in the months and years to come and how it develops in this climate of uncertainty will be key indicators of how this disempowering governance structure impacts the recovery and long-term disaster resilience of the territory.

### 4.4. Mexico City, 2017

On the 19th of September 2017, the centre of Mexico experienced a magnitude 7.1 earthquake. The epicentre of this earthquake was situated in the state of Puebla, approximately 150 km to the South-East of Mexico City. However, the impacts of this earthquake were disproportionately experienced in Mexico City. This was due to a combination of factors including the country’s proximity to major fault lines, Mexico City’s location on a former lake-bed, and the presence of unstable subsoils which amplify seismic waves. In terms of the death toll, official reports indicated that over 60 percent of more than 370 deaths occurred in Mexico City alone [113]. The concentration of earthquake-related fatalities in Mexico City could be due to the relatively dense urban population of close to nine million people. However, a combination of both death and population data highlights that the proportion of earthquake-related deaths in the city amounted to a highly disproportionate 62 percent of all earthquake-related deaths per capita across affected federal entities [114]. Mexico City was particularly vulnerable to this earthquake event as well as to future seismic events. In this case study, we highlight this vulnerability, together with the challenges of the early warning disaster management programmes in operation, to illustrate aspects of the Inverse Response Law.

Institutional arrangements prior to the Central Mexico earthquake and other recent earthquakes in this area help illustrate a partial but ongoing example of the Inverse Response Law. Following a much more severe, 8.1 magnitude earthquake in 1985, a similarly disproportionate death toll gave impetus to the development of an early earthquake warning system. The Sistema de Alerta Sísmica Mexicano (SASMEX) was originally developed through a public-private partnership to detect earthquakes originating in neighbouring areas of Mexico, before subsequent shockwaves would affect Mexico City. Reports received from agencies overseeing SASMEX indicate that multimedia displays and alert sirens are the principal, and most reliable, method to send an early earthquake alert to the Mexico City residents [115]. Although alerts have been sent via mobile internet networks, these alerts can be substantially delayed for a number of reasons, not least of all, due to overloaded mobile data networks [115]. This means that Mexico City inhabitants currently need to rely on fixed multimedia displays and siren placement for reliably accessing the benefits of SASMEX. Timely building evacuations and other potentially life-saving actions appear to be much less likely without the warnings delivered via access to this technological infrastructure.

However, anecdotal reports from Mexico City residents suggest that access via multi-media displays and siren coverage is far from universal throughout the city. More socio-economically disadvantaged areas of Mexico City such as Xochimilco do not form part of the siren and multimedia network. The siren network is highly visible in more wealthy parts of Mexico City, such as the suburb of Cuahtemoc. This apparent prioritisation of wealthier suburbs may have been justified by the presence of several large buildings on apparently unstable sub-soils. This combination of larger buildings and softer soils appears to have aggravated catastrophic collapses of built Cuatemoc infrastructure during the Central Mexico earthquake. However, the absence of a functioning and audible siren system in riverine areas of Mexico City such as Xochimilco remains a notable gap; one which coincides with and exacerbates high degrees of socioeconomic deprivation. The early warning system is funded by sub-national authorities, meaning that many seismically vulnerable poorer regions that are located outside of Mexico City are unable to afford this technology. The state of Chiapas, for example, where 16 people died is the poorest region in Mexico, and this region had no early warning system in place [116].

Since SASMEX’s inception in 1997 there is an enduring lack of evidence that timely warnings have been issued prior to any substantial earthquake event. The nearby origin of the 2017 Central Mexico Earthquake, for example, meant that early warning sirens sounded after major shaking was already being felt within Mexico City [115,117,118]. Furthermore, SASMEX has yet to be linked to evidence that protective actions, such as drop-cover-and-hold, are taken once a timely warning is received [115]. People in higher socio-economic groups are nonetheless more likely to comply with disaster preparedness and response measures [24,119]. Socio-economic differences in service provision, access and response to Mexico City’s early warning system therefore has the potential to increase inequitable outcomes between advantaged and disadvantaged groups within the same population.

Although aftershock activity was not a prominent feature of the 2017 Central Mexico earthquake, many affected residents appeared to be preparing for these and other earthquakes in the aftermath of this event [115]. Their activities and concerns form part of what has been referred to as a *cultura de prevención*, which roughly translates from Spanish to English to ‘a culture of prevention’. This term has also been compared to the wide-ranging concept of ‘seismic culture’, [115] in terms of promoting seismic awareness and the management of associated risks. Formal responses to this preparedness impetus, as provided and promoted by mandated government departments, exemplify another aspect of the Inverse Response Law. These governmental responses, in the form of building inspections and public information, had the potential to ameliorate or further exacerbate ongoing pre-earthquake vulnerabilities.

Without supplemental data concerning the distribution of inspections and the availability of suitably trained engineers, it is hard to fault hundreds of building inspections arranged by the Mexican government following the Central Mexico Earthquake. However, anecdotal reports from affected Mexico City residents and from Mexican engineers working on seismic safety suggest than state-funded building inspections have been focused on prior earthquake damage, rather than future seismic performance. Inspections among populations without resources to fund additional analyses are therefore unlikely to address seismic vulnerabilities built into structures from their initial construction. Nor are these inspections likely to address a range of options for strengthening buildings and correcting simple faults. Relevant vulnerabilities can include unsecured stairs wells, roofing, irregular building shapes and building levels which are not secured to one another [120].

This shortcoming may have been due to the need to prioritise a large number of damaged buildings, under certain financial constraints on public funding. Public information nonetheless appears to have worsened this aspect of inverse response and hazardous implications following the Central Mexico earthquake. Official advice from the Centro Nacional de Prevención de Desastres (CENAPRED), the official research centre advising emergency management throughout Mexico, provided a further example of the Inverse Response Law. Principal advice issued by CENAPRED during the first few weeks following the earthquake was to identify each building’s zones for safely sheltering during an earthquake [121]. This CENAPRED advice was accompanied by a recommendation that qualified architects or engineers should be contracted to identify each safe zone but lacked reference to any government funding for such contracts. Populations that are socioeconomically vulnerable are therefore unlikely to access information regarding safe sheltering, even though it is required to prevent further earthquake related deaths, injuries and relevant threats to livelihoods of affected populations. In sum, there appears to have been a lack of access to seismic safety and protective action information for populations in need both prior to, and following, the Central Mexico earthquake.

On a larger scale, the SASMEX system provides an example of neglecting disaster management needs, in favour of profits. This appears to replicate observations supporting the Inverse Care Law, because a functioning early earthquake warning system will ideally provide timely and clear warnings, linked to effective and realistic protective actions [115]. However, as part of the overall management of seismic risk in Mexico City, current SASMEX implementation does not appear to provide these benefits to many vulnerable parts of the population, including the residents of Xochimilco. The current SASMEX system may even be distracting residents from wider disaster management issues such as the apparent privatisation of more pro-active and preventative building inspections. In the meantime, public-private partnerships constituting SASMEX operations continue to receive the state funding that has ensured the profitability of private business entities, such as MDreieck, operating behind the scenes [122].

Given the unproven status of early earthquake warning in Mexico City, the broader ambit of disaster resilience appears to be particularly important. It should be noted that Mexico City has received substantial funding and in-kind support as part of the Rockefeller Foundation`s 100 Resilient Cities programme. This programme has been contributing two million USD to the promotion of measures to “help reduce social inequality and vulnerability, and produce a safer, fairer, and more equitable society” [123] (p. 6). However, as noted by Béné, Godfrey Wood, Newsham and Davies [124], issues concerning vulnerability can often be obscured by the concept of resilience. In the case of the Mexico City’s 100 Resilient Cities initiative, programme administrators seemed unable to systematically consult with vulnerable populations, when conducting their initial assessment of urban resilience [125]. This reasoning prompted Huggins and others [125] to focus on consulting with populations that were definitively vulnerable to earthquake, flooding, and socioeconomic risks at the inception of the 100 Resilient Cities programme in Colima City, Mexico. Time will tell if this approach, apparently much more feasible with a much smaller population compared to Mexico City, helps to mitigate inverse response dynamics as the Colima iteration of the 100 Resilient Cities programme progresses.

## 5. Discussion—Synergies across Examples and Relevance to Theory

The insights on disasters and the responses they evoke which the Inverse Care Law, and the Inverse Response Law, expose centre on spreading risk unevenly. That is, intentionally concentrating risk among the most vulnerable in society. Indeed this intentionality should probably constitute the first point of any sociological analysis of disaster, even though it predates whatever natural or man-made event that triggered it. In this sense disasters are socially constructed, they overwhelmingly impact the most vulnerable in society because of previous decisions that have left some at greater risk (as in the placement of early warning systems in Mexico City). This is not to say that some disasters do not entail a magnitude of destruction so that all members of society are impacted, but it does stress that even in this context the most vulnerable will be impacted the most. A second point is that the responses to disasters do not reverse the policies of neglect that prefigured or mediated, and in some cases (like the Grenfell Tower fire) were proximate to catastrophes; quite the opposite. Disaster response extends disaster construction, even though their presentation typically suggests care and unconstrained resourcing. There are several factors at play here. If we understand disaster response as an extension of the policies and dynamics that resulted in the uneven distribution of risk, the details of which are effectively the minutiae of disaster construction, then the primary purpose of any response is to validate those earlier responses. The heroics of accident and emergency professions, the role of volunteers, the statements of sympathy and support from politicians are in effect justifications of the previous policies. At the point of disaster the state, and it is ultimately the bourgeois state, is patently treating all its citizens the same (women and children first, of course). The response to disaster (firefighting, sand bags, soup kitchens, identifying bodies) merges from the very start with what journalists might call a cover up.

In the context where disasters inevitably provide a disruption of everyday norms wherein new sorts of question might (for a short time) be asked, the response to disaster prefigures a normative reply. Hence the Inverse Response Law is inverse in several ways. In the everyday post-disaster, not all survivors are the same (as in the case of Kaikōura). Those who were vulnerable before the disaster event are likely to be less resilient in the aftermath. Insofar as rescue and response efforts are interactive vulnerable survivors have significantly less capacity for claims-making and (with some caveats) social solidarity. Further, the rescuers (ultimately, the state) does not treat all survivors the same. The social hierarchy that generated the uneven distribution of social risk in the first instance remains in place, and because of the need to justify that social inequality (at least through the news cycle and beyond into any formal enquires), it is repeated and amplified. The result is stigmatisation, where vulnerable survivors, such as those in Puerto Rico, tend to be portrayed as bad survivors (looters, opportunists, refusing to follow instructions, benefitting undeservedly from the relief effort) and even co-creators of their misfortune (choosing to live in unsafe conditions, willfully unprepared, lazy, stupid, superstitious). A third aspect of disaster and disaster response represents something of a counter-intuitive to the original formulation by Tudor Hart. The political raison d’être of the inverse law of care was integrative. Tudor Hart argued that better (complete) integration of patients into the health system would reduce vulnerability. However, the social construction of disasters suggests that there are considerable benefits for vulnerable survivors if they are not fully integrated: That is, not being fully integrated into a socio-economic system which intentionally renders them vulnerable and likely to be stigmatised post-disaster. The case studies presented illustrate how the Inverse Response Law is a social product and anticipatory in a structural sense. Its impacts are the result of multiple and at times cross-cutting policies whose end result is to draw attention away from the social conditions that contribute to disaster vulnerability and to reduce the entitlement of categories of social actors to support. Though situated in diverse contexts, the case studies presented in this article constitute mirror exemplars of the ways in which disaster responses extend disaster construction through contemporaneously undermining social solidarity, providing inequitable support and perpetuating disaster risks to the most vulnerable in society.

## 6. Conclusions

According to Alexander [5] ‘[t]he accumulation of a body of theory legitimizes a discipline and helps it to become mature’ (p. 8). In this regard, the more established fields of sociology and public health have much to offer to the fledging disaster studies and emergency management disciplines. The Inverse Response Law blends the broader theoretical perspectives of sociology with the principles within the Inverse Care Law from public health. In the case studies presented in this article different ideas from sociology are drawn upon in order to illustrate aspects of the Inverse Response Law. The Grenfell Tower example exemplifies the way in which economic restructuring, and the withdrawal of the welfare state, transfers risks to the most vulnerable. How social capital influences access to services in the weeks and months following a disaster is considered in the case study on Kaikōura. Hurricane María illustrates the role of colonial power as well as inequality in shaping disaster risk and response. While the distribution of seismic risk through institutional arrangements that affect public access to early warning systems is the focus of attention in Mexico City. Taken together, the case studies draw attention to the structural conditions that influence the social patterning of vulnerability as well as disaster risk. In each case, vulnerability can be traced back to deliberate decisions that were made by policy makers—in this regard the many negative human impacts of the disasters are social constructions.

In a commentary on the Inverse Care Law Tudor Hart [12] expressed disappointment at how inequities in access to health care has been prioritised over his arguments about how market forces influence the provision of medical services over time and space. Attention to the geographical distribution of services as well as the role of economics in shaping vulnerability in this article is a response to Tudor Hart’s concerns about how his ideas have been taken up and used within public health. Structural approaches to disaster risk reduction which draw upon ideas from public health, as well as the explanatory of potential social theory, have the ability to improve outcomes through demonstrating the need for environments, policies and regulations that support equity and resilience.
